# An Unusual Case of Upper Digestive Bleeding—Metastatic Amelanotic Melanoma: Case Report and Literature Review

**DOI:** 10.3390/life16030469

**Published:** 2026-03-13

**Authors:** Mihaela Dranga, Cristina Cijevschi Prelipcean, Otilia Nedelciuc, Alina-Ecaterina Jucan, Georgiana-Elena Sârbu, Atodiresei Carmen, Iolanda Valentina Popa, Roxana Nemțeanu, Irina Ciortescu, Victor Ianole, Catalina Mihai

**Affiliations:** 1 Department of Medical Science, Grigore T. Popa University of Medicine and Pharmacy, University Street 16, 700115 Iasi, Romania; mihaela_dra@yahoo.com (M.D.); ghiata.alina.ecaterina@gmail.com (A.-E.J.); georgiana_ciobanu23@yahoo.com (G.-E.S.); carmen.atodiresei@yahoo.com (A.C.); iolanda-valentina.g.popa@umfiasi.ro (I.V.P.); maxim_roxxana@yahoo.com (R.N.);; 2 Institute of Gastroenterology and Hepatology, Sf. Spiridon Emergency Clinical Hospital Iasi, Bulevardul Independenței nr. 1, 700111 Iasi, Romania; cristinacijevschi@yahoo.com; 3 Department of Internal Medicine, Grigore T. Popa University of Medicine and Pharmacy, University Street 16, 700115 Iasi, Romania; 4 Department of Pathology, Grigore T. Popa University of Medicine and Pharmacy, University Street 16, 700115 Iasi, Romania; victor.ianole@umfiasi.ro

**Keywords:** amelanotic melanoma, gastrointestinal metastasis, endoscopy

## Abstract

Metastatic melanoma is one of the most common malignancies associated with the spread of the primary tumor. The primary site is usually the skin or the eyes. The most frequent site of metastases is the gastrointestinal tract, accounting for 60% of cases at autopsy. In 2% of patients, metastases occur without a detectable primary tumor. We present a rare case of upper digestive bleeding caused by multiple gastrointestinal tract metastases from an amelanotic melanoma. This case report describes a 65-year-old male who arrived at the emergency department after experiencing an episode of upper gastrointestinal bleeding (melena). One week prior to admission, he had been treated with nonsteroidal anti-inflammatory drugs for lower back pain due to L4–L5 disc herniation. Upper digestive endoscopy revealed multiple polypoid masses in the stomach and duodenum, and capsule endoscopy showed additional lesions in the small bowel. Histopathological examination confirmed the diagnosis: metastases from an amelanotic malignant melanoma. Abdominal and cranial computed tomography scans revealed multiple secondary lesions in the brain, gallbladder, retroperitoneal area, gastrointestinal tract, and peritoneum. Localized radiotherapy was applied to the cerebral metastasis, and systemic chemotherapy with dacarbazine was initiated, resulting in a partial clinical response. Unfortunately, the disease progressed, and the patient died one month after diagnosis. Metastatic melanoma of the gastrointestinal tract is an exceedingly rare cause of upper digestive bleeding.

## 1. Introduction

Malignant melanoma is one of the most aggressive cancers, capable of spreading to any organ. The primary site is usually in the tissue where melanocytes are located, such as the skin, eyes, meninges, rectum, sigmoid colon, and, more rarely, in the esophagus, stomach, and small bowel. The gastrointestinal tract is the most common site of metastases, accounting for 60% of patients at autopsy in one center [1]. Only 4% of metastases are detected during the patient’s lifetime because they often do not show specific symptoms. In 2% of patients, metastases are present without a detectable primary tumor [2].

GI localization of metastasis is often silent, with symptoms typically arising from complications such as perforation, hematemesis, and melena. Metastases are generally pigmented, although they can be amelanotic, as in our case [3].

In the literature, few cases of metastatic amelanotic melanoma have been described [4], and even fewer with metastases to the entire digestive tract. Usually, the intestinal metastasis reported in the literature was solitary [5]. In this case report, we present a rare case of amelanotic malignant melanoma that has disseminated metastases, both beyond the digestive system and throughout the entire digestive tract.

## 2. Case Report

A 65-year-old male patient presented to the Emergency Department (ED) after an episode of upper GI bleeding (melena) and was admitted to the Gastroenterology Department. A week before his admission, he had been treated with nonsteroidal anti-inflammatory drugs (NSAIDs) for lower back pain (L4–L5 disk herniation). His personal history included hypertension and dyslipidemia with an unremarkable family history. He was an occasional drinker, a non-smoker, and did not take any chronic medications. On physical examination, we noted skin pallor, tachycardia, an enlarged lymph node in the right axilla, and hepatomegaly. Additionally, the patient reported seeing phosphenes and photopsia.

The laboratory findings indicated iron deficiency anemia and thrombocytosis. Hb (Hemoglobin) level is 8.1 g/dL (normal range 12–16 g/dL), Ht (hematocrit) is 26% (normal range 35–47%), MCV (mean corpusculare volume) is 75.6 fl (normal range 78–96 fl), MCH (mean corpuscular hemoglobin) is 23.5 pg/cell (normal range 27–34 pg/cell), MCHC (mean corpuscular hemoglobin concentration) is 31.5%(normal range 31–36%), PLT (platelet count) is 568.000/mm^3^ (normal range 150,000–400,000/mm^3^), and iron level is 20 μg/dL (normal range 50–120 μg/dL).

**Figure 1:**
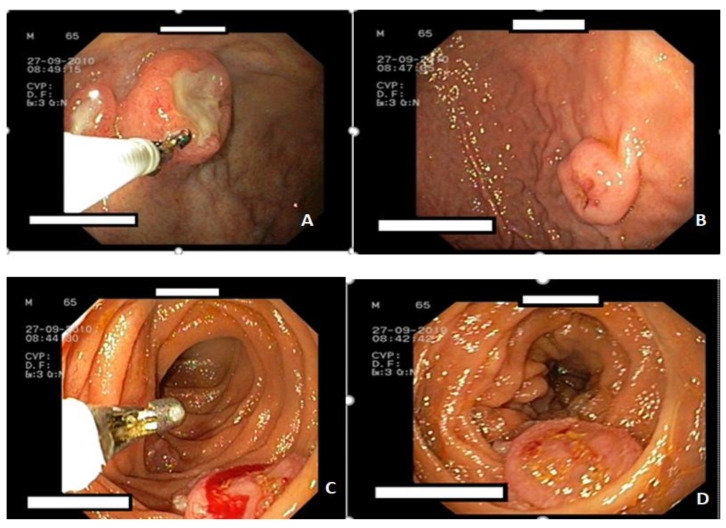
(**A**–**D**). Upper GI endoscopy**:** Oval lesions with a depressed center in the stomach and duodenum. (**A**). Endoscopic image of the stomach showing a polypoid lesion on the anterior wall, centrally ulcerated. (**B**). Endoscopic image of the stomach showing a polypoid lesion on the posterior wall, with central excavation. (**C**). Endoscopic image of the second part of the duodenum showing a polypoid lesion with biopsy. (**D**). Endoscopic image of the second part of the duodenum showing a polypoid lesion.

The upper endoscopy revealed multiple elevated oval lesions, some ulcerated, in the gastric body, antrum, and duodenum (Figure 1). The surrounding mucosa showed no abnormalities.

**Figure 2:**
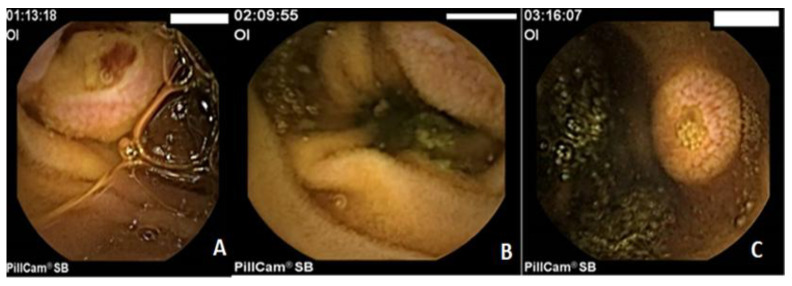
(**A**–**C**). Capsule endoscopy revealed multiple protruding, oval lesions with central ulcers and adherent clots in the jejunum. The histopathological examination of the gastric antral biopsy showed an ulcerated mucosa with tumor infiltration by spindle, atypical cohesive cells (Figure 3A). Immunohistochemistry indicated that the tumor cells tested positive for melanoma markers S100 (Figure 3B) and HMB45 (Figure 3C), but were negative for Cytokeratin AE1/AE3 (Figure 3D), synaptophysin, and Chromogranin A, confirming the diagnosis of melanoma.

The capsule endoscopy revealed multiple protruding, oval lesions measuring between 0.5 and 2 cm, covered by normal mucosa, with several being ulcerated or showing adherent clots. These lesions were found in the duodenum and extended throughout the jejunum (Figure 2). The colonoscopy showed no additional lesions.

**Figure 3:**
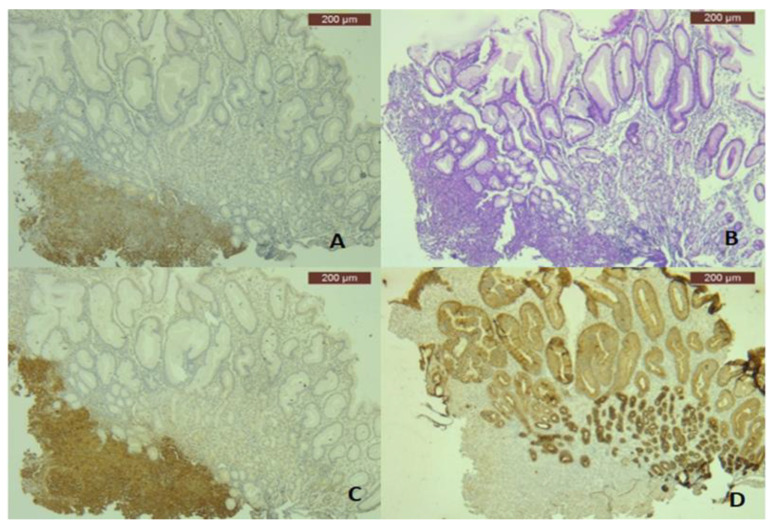
Microscopical features of amelanotic melanoma: tumor cells infiltrate the antral mucosa (**A**) (HE, ×50) and are positive for S100 (**B**) (IHC, anti-S100 Ab, ×50) and HMB45 (IHC, anti-HMB45, ×50) (**C**), but are negative for Cytokeratin AE1/AE3, which is positive in the normal adjacent gastric epithelium (IHC, anti-CK AE1/3, ×50) (**D**).

The histopathological examination of the antral gastric biopsy revealed an ulcerated mucosa with tumor infiltration characterized by spindle, atypical, cohesive cells (Figure 3A). Immunohistochemistry demonstrated that the tumor cells were positive for melanoma markers S100 (Figure 3B) and HMB45 (Figure 3C), but negative for Cytokeratin AE1/AE3 (Figure 3D), synaptophysin, and Chromogranin A, confirming the diagnosis of melanoma.

Considering the histological diagnosis of melanoma, additional investigations were performed to identify other sites of the tumor. Therefore, the patient underwent dermatological and ophthalmological evaluation, as well as cranio-thoraco-abdominal contrast-enhanced computed tomography.

The primary site was not identified.

**Figure 4:**
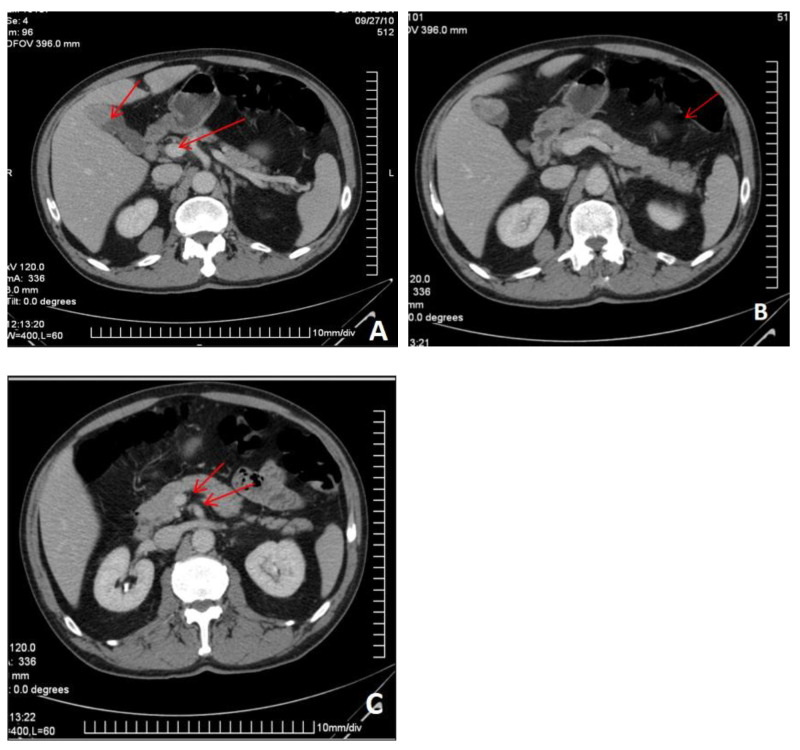
Axial enhanced abdominal portal-venous phase CT images showing a gallbladder mass (**A**), a pancreatic type enhancing mass (**B**), and retroperitoneal lesions (**C**), all highly suggestive of metastasis (arrows).

The abdominal CT revealed multiple secondary lesions in the gallbladder, retroperitoneal area, GI tract, and peritoneum. Without a histopathological examination, the primary or secondary nature of these lesions could not be established (Figure 4).

**Figure 5:**
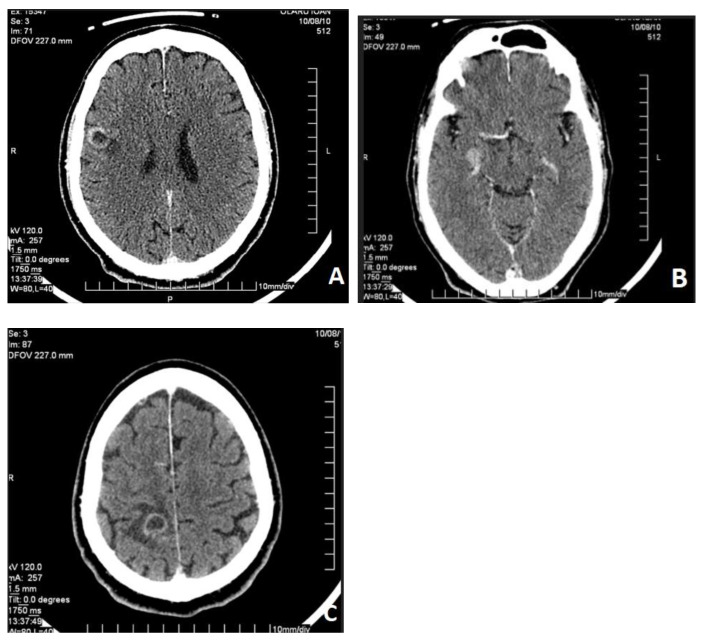
Axial postcontrast supratentorial cranial CT images show intraneural lesions with visible annular enhancement ((**A**,**C**), in the right frontal and parietal lobes) associated with perilesional edema, respectively, a homogeneously enhanced lesion ((**B**) in the right cerebellar peduncle).

Regarding the neurological symptoms (phosphenes and photopsy), a CT scan was performed, revealing supratentorial, mainly in the right cerebral hemisphere, solid masses that are hyperdense in the native phase and heterogeneous due to necrosis, with slight contrast enhancement in the parenchymal phase. After contrast agent administration, the homogeneous or ring-shaped lesions in the white matter were surrounded by perilesional edema, along with supratentorial secondary disseminations (Figure 5).

Localized radiotherapy for the cerebral metastasis and systemic chemotherapy with dacarbazine were initiated, at a dose of 250 mg/m^2^ on days 1–5. Only one cycle of dacarbazine was administered, resulting in a partial clinical response. Unfortunately, the patient ultimately passed away one month after diagnosis due to a massive hemorrhagic stroke.

## 3. Discussion

There are some hypotheses suggesting that GI melanomas, especially those of unknown origin, originate from a cutaneous melanoma with regression [6]. The differential diagnosis to distinguish between a primary and secondary melanoma of the GI tract can be challenging. For the diagnosis of a primary malignant melanoma, the following criteria must be met: 1. Exclusion of other lesion sites; 2. The absence of other skin tumors or extra digestive lesions; and 3. The absence of other intestinal lesions [7]. In this case, none of the criteria for primary malignant melanoma were fulfilled.

Metastatic melanoma is one of the most common malignancies associated with the spread of the primary tumor [8]. In a review from Memorial Sloan Kettering Cancer Center, the authors reported that the incidences of metastatic sites found at autopsy were: liver, 68%; small bowel, 58%; colon, 22%; stomach, 20%; duodenum, 12%; rectum, 5%; esophagus, 4%; and anus, 1% [9]. Autopsies of 216 patients with advanced malignant melanoma revealed that the most common site for secondary spread was the lungs, followed by the gastrointestinal (GI) tract [10]. The prevalence of GI metastases was 43.5%. Multiple organ metastases were common (95%). The distribution of metastatic lesions in the abdomen included: liver, 58.3%; peritoneum, 42.6%; pancreas, 37.5% [11]; small bowel, 35.6%; spleen, 30.6%; colon, 28.2%; stomach, 22.7%; oral cavity and esophagus, 9.3%; and biliary tract, 8.8% [10]. In a recent study, La Selva et al. identified only 34 patients with primary and metastatic melanomas of the GI tract from 1996 to 2018. The cases included 7 primary GI melanomas and 27 metastatic melanomas involving the digestive tract and gallbladder [5].

Typical metastatic malignant melanomas are often asymptomatic. The clinical signs are usually nonspecific, mimicking a gastrointestinal tumor, and may include fatigue, weakness, constipation, or tenesmus. However, hematemesis, melena, or intussusception can sometimes be the initial symptoms [12]. A less common presentation is bloody stools, especially when the primary tumor or metastasis is located in the anorectal region [13].

Our patient presented with upper digestive bleeding, considered to be secondary to NSAID intake. There are only a few cases of metastatic malignant melanoma in the literature presenting with upper digestive bleeding or iron deficiency anemia [14,15].

Diagnosis of metastatic melanoma is usually confirmed through endoscopic evaluation and radiographic contrast procedures, including CT [8]. Upper GI endoscopy may identify gastric metastasis as black-pigmented ulcers, multiple mucosal or submucosal nodules, or polypoid lesions [16].

Suganuma et al. described a submucosal tumor-like elevated lesion with a depression on the posterior wall of the middle gastric body. It was diagnosed as a stomach metastasis from primary small intestinal malignant melanoma [17]. In our case, the presence of protruding, discoidal lesions, some ulcerated, without melanin pigment, raised differential diagnosis challenges.

The definite diagnosis was established by histopathological examination. It is difficult to accurately diagnose amelanotic malignant melanoma because there are usually no melanin pigments. To our knowledge, there are very few cases of amelanotic multiple metastases cited in the literature [18,19]. Therefore, when the disease is not suspected, pathological immunostaining is not performed, so some amelanotic melanomas remain undiagnosed. In our case, we performed immunostaining assays, such as S100 proteins and HMB-45 antibody, and the biopsy samples were positive for HMB-45 and S100 proteins despite the absence of melanin pigments.

Another peculiarity of our case was that, despite multiple abdominal and brain metastases, the primary tumor site was not identified. CT examination revealed supratentorial lesions that were interpreted as secondary disseminations. Some authors have also described primary cerebral cases of multifocal melanoma. Cao described a multifocal primary amelanotic meningeal melanoma in the left temporal and right frontal lobes [20]. Another case of amelanotic meningeal melanoma with leptomeningeal dissemination was recently described by Zhang et al. [21]. Functional magnetic resonance examinations raised the question of malignant melanoma, but the final diagnosis was made by immunohistochemistry [21].

Another rare metastatic site in this case is the gallbladder. Gallbladder involvement is uncommon and usually occurs in the context of diffuse metastatic disease [22].

Unfortunately, the patient’s family does not want an autopsy to be performed in this case to determine if one of the lesions was primary.

The treatment for malignant melanoma involves systemic chemotherapy with Dacarbazine. In cases of complications, such as hemorrhage, endoscopic procedures—like endoscopic hemostasis—can be employed [23]. Recent studies have shown that immunotherapy using the monoclonal anti-CTLA4 antibody may be effective in treating metastatic melanoma [24]. The prognosis is poor, with an average survival of only 4 to 6 months. Our patient received Dacarbazine chemotherapy and was evaluated for immunotherapy, but unfortunately, he died one month after diagnosis due to a massive hemorrhagic stroke.

## 4. Conclusions

In conclusion, the gastrointestinal tract can be a site of amelanotic malignant melanoma metastasis, and physicians should be aware of melanoma involvement even in patients who are asymptomatic or have few symptoms. Metastatic GI melanoma is a rare cause of upper gastrointestinal bleeding. Endoscopists should pay close attention to lesions observed during endoscopy, even when there is no history of melanoma. Endoscopic and histological differential diagnosis is challenging in amelanotic melanoma. The primary tumor often develops silently, and the diagnosis is usually made at the stage of widespread metastases, which carries a guarded prognosis.

## Data Availability

The data presented in this study are available on request from the corresponding author. The data are not publicly available due to privacy.
